# Natriuretic Peptide and Clinical Evaluation in the Diagnosis of Heart Failure Hemodynamic Profile: Comparison with Tissue Doppler Echocardiography

**DOI:** 10.5935/abc.20180046

**Published:** 2018-03

**Authors:** Gustavo Luiz Gouvêa de Almeida Junior, Nadine Clausell, Marcelo Iorio Garcia, Roberto Esporcatte, Fernando Oswaldo Dias Rangel, Ricardo Mourilhe Rocha, Luis Beck-da-Silva, Fabricio Braga da Silva, Paula de Castro Carvalho Gorgulho, Sergio Salles Xavier

**Affiliations:** 1 Casa de Saúde São José, Rio de Janeiro, RJ - Brazil; 2 Hospital Pro-Cardíaco, Rio de Janeiro, RJ - Brazil; 3 Hospital Universitário Clementino Fraga Filho - Universidade Federal do Rio de Janeiro (UFRJ), Rio de Janeiro, RJ - Brazil; 4 Hospital de Clínicas de Porto Alegre - Universidade Federal do Rio Grande do Sul, Porto Alegre, RS - Brazil

**Keywords:** Heart Failure, Natriuretic Peptide, Brain, Hemodynamics, Ventricular Function, Left, Echocardiography, Doppler

## Abstract

**Background:**

Physical examination and B-type natriuretic peptide (BNP) have been used to estimate hemodynamics and tailor therapy of acute decompensated heart failure (ADHF) patients. However, correlation between these parameters and left ventricular filling pressures is controversial.

**Objective:**

This study was designed to evaluate the diagnostic accuracy of physical examination, chest radiography (CR) and BNP in estimating left atrial pressure (LAP) as assessed by tissue Doppler echocardiogram.

**Methods:**

Patients admitted with ADHF were prospectively assessed. Diagnostic characteristics of physical signs of heart failure, CR and BNP in predicting elevation (> 15 mm Hg) of LAP, alone or combined, were calculated. Spearman test was used to analyze the correlation between non-normal distribution variables. The level of significance was 5%.

**Results:**

Forty-three patients were included, with mean age of 69.9 ± 11.1years, left ventricular ejection fraction of 25 ± 8.0%, and BNP of 1057 ± 1024.21 pg/mL. Individually, all clinical, CR or BNP parameters had a poor performance in predicting LAP ≥ 15 mm Hg. A clinical score of congestion had the poorest performance [area under the receiver operating characteristic curve (AUC) 0.53], followed by clinical score + CR (AUC 0.60), clinical score + CR + BNP > 400 pg/mL (AUC 0.62), and clinical score + CR + BNP > 1000 pg/mL (AUC 0.66).

**Conclusion:**

Physical examination, CR and BNP had a poor performance in predicting a LAP ≥ 15 mm Hg. Using these parameters alone or in combination may lead to inaccurate estimation of hemodynamics.

## Introduction

Clinical evaluation of patients with acute decompensated heart failure (ADHF) based only on physical examination has proved to be inadequate for both assessment of left ventricular (LV) function (systolic versus diastolic dysfunction)^1,2^ and estimation of patient’s hemodynamic status.^3^ Precise determination of LV filling pressures is critical to the proper treatment of patients with ADHF, since congestion is the main determinant of symptoms, hospitalization, and prognosis.^4-7^ Additional assessment using both invasive^8^ and noninvasive tools may be useful, as it adds important information potentially contributing to tailored management.

Echocardiography has proven its usefulness in assessing the hemodynamic status of patients with ADHF, especially after the advent of new techniques, such as tissue Doppler imaging.^9^ The so-called “hemodynamic echocardiogram” may help physicians to detect congestion.^10^ Several studies have shown that echocardiographic-derived hemodynamic parameters correlate significantly with those obtained by right heart catheterization.^11^

Elevated levels of B-type natriuretic peptide (BNP) reflect increased LV filling pressures, secondary to myocyte stretch, due to volume or pressure overload.^12,13^ Whether the association of BNP values adds diagnostic accuracy to the standard clinical assessment in estimating patient’s hemodynamic status remains unknown. In this study we tested the hypothesis that BNP values add diagnostic accuracy to physical examination in detecting congestion in patients with ADHF, using echocardiogram-derived hemodynamic assessment as a reference method for comparison.

## Methods

### Sample studied

A prospective convenience sample of patients admitted to the emergency department or coronary care unit of three hospitals (one university-affiliated and two tertiary hospitals) due to ADHF was studied. The study was conducted according to the Declaration of Helsinki standards for human research. Institutional review boards approved the research protocol, and all participants provided written informed consent before enrollment.

### Inclusion criteria

Patients with ADHF due to LV systolic dysfunction, with LV ejection fraction (LVEF) <40% by Simpson's method, New York Heart Association (NYHA) functional class III or IV on admission and sinus rhythm were included within 24 hours of emergency care.

### Exclusion criteria

The exclusion criteria were as follows: ADHF due to acute coronary syndrome; echocardiographic window precluding adequate analysis of hemodynamic parameters; primary valve disease; mechanical prosthetic valve; single mitral flow pattern; and presence of cardiac pacing.

### Physical examination

The following physical findings were evaluated: jugular venous distension; hepatojugular reflux; hepatomegaly; ascites; lower extremity edema; third heart sound (S3); pulmonary rales; arterial blood pressure; and proportional pulse pressure. Patients were examined in a quiet emergency or critical care room. The jugular venous distension was evaluated with the patient sitting upright and the presence of a visible internal jugular vein above the clavicle was considered elevated. The hepatojugular reflux was tested in patients with no visible jugular vein, applying a firm right abdominal pressure. The liver was examined with the patient in recumbent position. Hepatomegaly was considered when the liver had more than 10cm of length, considering percussion technique initiating on the third intercostal space, along the midclavicular line. Liver palpation was the method of choice to assess the lower margin of the liver if it was palpable in the abdomen.

Patients with chest radiography showing any sign of congestion were considered to be congested. The radiological evaluation was done through the posterior-anterior and left lateral chest radiography. In cases of inability to take chest radiography in posterior-anterior and lateral positions, an anterior-posterior position with the patient sitting in bed was performed. The chest radiography was performed immediately prior to the echocardiogram.

### B-type natriuretic peptide assay

Simultaneously to echocardiography, blood sample was drawn for measurement of BNP. Samples were drawn in EDTA tubes and BNP was measured in whole blood, by immunofluorescence technique, using a commercially available kit (Triage ® BNP test of Biosite Inc., San Diego, CA, USA). All measurements were performed within 30 minutes of blood sampling. Patients with levels of BNP > 400 pg/mL were considered congested,^14^ and with levels of BNP < 200 pg/mL were considered “dry”.^15^

### Echocardiogram evaluation

All patients were submitted to a transthoracic echocardiography with tissue Doppler imaging (GE Vivid 7, Wauwatosa, WI, USA) within a maximum of 30 minutes after completion of the physical exam. In each center, only one examiner (the most experienced) performed all echocardiographic evaluations. Echocardiographic measurements were performed in a blinded manner: the examiner was unaware of the physical findings. Images were obtained from patients in the left lateral and recumbent position, and measurements followed the recommendations of the American Society of Echocardiography.^16^ All Doppler profiles were recorded in an apical 4-chamber view.

The estimated left atrial pressure (LAP) was calculated as follows: calculation of the E/E' ratio by measuring the intra-myocardial flow velocity with tissue Doppler. The early diastolic mitral annular velocity (E') was obtained by tissue Doppler in the LV lateral wall and in case of technical impossibility of obtaining the velocity in this wall, as in ischemic involvement, it was measured in the interventricular septum. At least three consecutive cardiac cycles were used and an average was used as the final result. This measurement, when combined with the trans-mitral flow obtained with pulsed Doppler in early diastole (E) results in the relationship E/E'. The LAP was then estimated by the formula: LAP: 1.24 x (E/E’) + 1.9. Indication of increased LV filling pressure was defined as LAP ≥ 15 mm Hg. Although patients with values below 15 mm Hg may have congestion, values ≥ 15 mm Hg have high specificity for increased LV filling pressure. Ejection fraction was evaluated through the Simpson’s method.

### Statistical analysis

Descriptive statistics were expressed as frequency (%) for categorical variables. For continuous variables data are presented as means ± standard deviation for normally distributed data or median and interquartile range (IQR) for non-normally distributed data. Measures of diagnostic performance (sensitivity, specificity, accuracy, positive and negative predictive values) were used to evaluate the diagnostic utility of physical exam signs of heart failure and/or BNP in predicting LAP ≥ 15 mm Hg (defined as indication of increased LV filling pressure).

The Spearman test was used to analyze the correlation between non-normal distribution variables. The level of significance was 5%.

To determine the best cut-off value for BNP to estimate elevation in LAP, a receiver operating characteristic (ROC) curve was constructed. A clinical score (CS) was built by giving 1 point to each positive sign of decompensated heart failure (elevated jugular venous distension, hepatojugular reflux, hepatomegaly, pulmonary rales or edema). Patients with ≥ 2 points were considered with a positive CS, according to analysis of ROC curve. To evaluate the capacity of physical exam and noninvasive diagnostic tests for the prediction of elevated LAP (LAP ≥ 15 mm Hg), separate models were built using combination of CS, CS + chest radiography, CS + chest radiography + BNP > 400 pg/mL, and finally CS + chest radiography + BNP > 1000 pg/mL (based on optimal cut-off point of BNP). Each of these diagnostic tests was dichotomized and compared to determine the incremental predictive value. Statistical analyses were performed using SPSSÒ (SPSS Inc, Chicago, IL, USA).

## Results

### Patients characteristics

Forty-three patients were included in the study. Patients were predominantly male (75%), elderly (69.9 ± 11.1 years) and had ADHF of ischemic etiology (65%). The mean serum creatinine was 1.3 ± 0.4 mg/dL, and the mean BNP was 1057 pg/mL ± 1024 pg/mL. Table 1 shows clinical and demographic characteristics of patients. All patients were in NYHA functional class III (10.7%) or IV (89.3%), with a mean LVEF of 25% ± 8.0%.

**Table 1:** Clinical and demographic characteristics of the patients

Characteristics	
n	43
Age (years)	69.9 ± 11.1
Gender (male %)	76
Weight	75.3 ± 17.1
Body mass index (kg/m^2^)	26.55.2
**Etiology**	
Ischemic	28 (65.1)
Idiopathic	7 (16.2)
Hypertensive	3 (6.9)
Valvular	4 (9.3)
Others	1 (2.3)
Left ventricular ejection fraction (%)	25.6 ± 8.0
B-type natriuretic peptide (pg/mL)	1057.39 ± 1024.21
Urea (mg/dL)	60.7 ± 23.4
Creatinine (mg/dL)	1.3 ± 0.4
Sodium (mEq/L)	135.9 ± 5.4
Potassium (mEq/L)	4.1 ± 0.5
Hemoglobin (g/dL)	11.8 ± 1.9

Nine patients had LAP < 15 mm Hg as assessed by echocardiogram. The most frequent sign of decompensation was the presence of rales (27 patients), followed by S3 (19 patients), edema, hepatomegaly and hepatojugular reflux (12 patients each). Prevalence of all clinical signs is show in Table 2.

**Table 2:** Frequency of physical signs of heart failure decompensation

Physical Sign	Frequency (n°)
S3	19
PJVD	8
HJR	12
Rales	27
Edema	12
Ascites	1
Hepatomegaly	12

S3: third heart sound; PJVD: pathologic jugular venous distension; HJR: hepatojugular reflux.

### Accuracy of clinical signs to predict increased LV filling pressures

Elevated jugular venous pressure was the most specific (88%) clinical sign to predict LAP ≥ 15 mm Hg, and rales were the least specific (33%). Accuracy of each sign to predict LAP ≥ 15 mm Hg is shown in Table 3. Combining any two signs of congestion has the best accuracy to predict elevation in LAP, according to the ROC curve.

**Table 3:** Diagnostic characteristics of clinical signs to predict left atrial pressure ≥ 15 mm Hg

	Sensitivity	Specificity	PPV	NPV	Accuracy
S3	44	55	79	20	46
PJVD	20	88	87	22	34
HJR	29	77	83	22	39
Edema	29	77	83	22	39
Hepatomegaly	29	77	83	22	39
Rales	61	33	77	18	55

S3: third heart sound; PJVD: pathologic jugular venous distension; HJR: hepatojugular reflux, PPV: positive predictive value, NPV: negative predictive value.

### Accuracy of chest radiography and BNP to predict increased LV filling pressures

**Figure 1:**
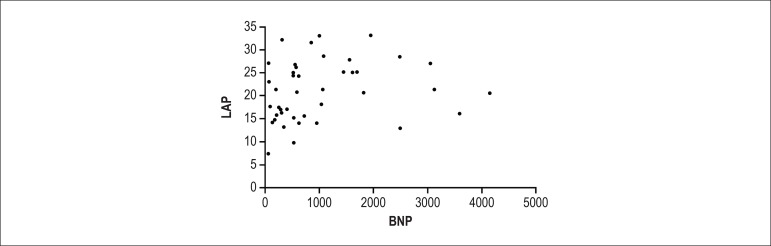
Correlation between left atrial pressure (LAP) and B-type natriuretic peptide (BNP). r = 0.3 (p = 0.046).

Levels of BNP > 400 pg/mL had a suboptimal diagnostic capability to estimate congestion. Figure 1 illustrates the poor correlation between BNP and echocardiographically assessed LAP.

In fact, chest radiography showed a slightly better accuracy than BNP levels to predict congestion. Table 4 shows the performance of these variables to predict LAP ≥ 15 mm Hg. We constructed a ROC curve to estimate the best cut-off point of BNP to predict elevation of LA filling pressure. Levels of BNP > 1000 pg/mL showed a specificity of 88% and a positive predictive value of 93% to predict congestion, but this cut-off loses sensitivity (44% vs 73%) and accuracy (53% vs 67%) when compared with a value > 400 pg/mL (Table 4).

**Table 4:** Diagnostic characteristics of the B-type natriuretic peptide (BNP) and chest radiography to predict left atrial pressure ≥ 15 mm Hg

	Sensitivity	Specificity	PPV	NPV	Accuracy
BNP > 400	73	44	83	30	67
BNP >1000	44	88	93	29	53
Chest radiography	79	44	84	36	72

PPV: positive predictive value; NPV: negative predictive value.

### Combinations of clinical signs, chest radiography and BNP to predict increased LV filling pressures

Table 5 depicts the diagnostic characteristics of the CS alone, CS plus chest radiography and these two plus BNP > 400 pg/mL to predict LAP ≥15 mm Hg. Incremental accuracy was observed when progressively combining these parameters. The three parameters combined achieved a sensitivity of 91% and a positive predictive value of 81% to detect a LAP ≥ 15 mm Hg.

**Table 5:** Diagnostic characteristics of clinical score, chest radiograph (CR), B-type natriuretic peptide (BNP) and all combined to predict left atrial pressure ≥ 15 mm Hg

	Sensitivity	Specificity	PPV	NPV	Accuracy
CS+	64	33	78	20	58
CS+ plus CR	82	33	82	33	72
CS+ plus CR plus BNP > 400	91	22	81	40	76

CS+: positive clinical score; PPV: positive predictive value; NPV: negative predictive value.

### Diagnostic performances of combined clinical tools

**Figure 2:**
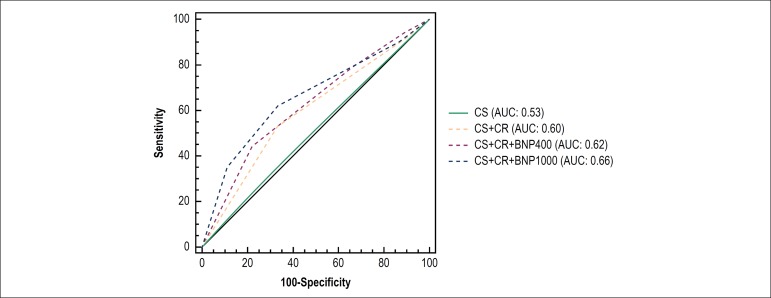
Receiver operator characteristics curves for estimating left atrial pressure ≥ 15 mm Hg. Estimates were based on dichotomized variables. CS: clinical score; CR: chest radiography; BNP: B-type natriuretic peptide.

Accuracy of CS and its combinations with chest radiography and BNP with cut-off values of 400 pg/mL or 1000 pg/mL are illustrated in Figure 2. Combinations of CS with chest radiography (AUC 0.60) and BNP > 400 pg/mL (AUC 0.62) did not improve the ability to discriminate between low or high LAP. Combination with levels of BNP > 1000 pg/mL improved only modestly (AUC 0.66).

## Discussion

In this study, we have assessed the diagnostic accuracy of heart failure clinical signs to predict elevation of cardiac filling pressures as derived from echocardiogram-based parameters. Additionally, we have combined information from clinical signs and chest radiography regarding congestion and finally added the BNP value in order to augment the diagnostic accuracy in assessing congestion. This strategy reflects a “real world” practice to clinically evaluate the hemodynamics of ADHF patients, and we compared this clinical approach with objective measurements of hemodynamics derived from tissue Doppler echocardiogram. We have shown that a CS of congestion, chest radiography and BNP, alone or in combination do not accurately predict elevation of LAP.

### Clinical findings in ADHF

#### Jugular venous pressure

The jugular venous pressure is the most important and probably the only physical examination sign that is relatively accurate in estimating ventricular filling pressures.^17^ In a study with 35 patients in a critical care unit, the jugular venous pressure was accurate in estimating low or high filling pressures.^18^ In another study, after evaluating a thousand patients referred for cardiac transplantation, the authors observed that estimated right atrial pressure below or above 10 was concordant with a pulmonary capillary wedge pressure (PCWP) below or above 22 mm Hg in 79% of patients.^19^ Other studies also have shown prognostic information about elevated jugular venous pressure in patients with heart failure. Its presence was associated with adverse outcome, including progression of heart failure, even after adjustment for other prognostic factors.^20^ But several factors limit its power in predicting filling pressures. There is not a universal method to estimate the jugular venous pressure. Controversy exists regarding the position (sitting upright or semirecumbent position of 30-45º), the jugular vein being used (internal x external), and the technique of measurement (vertically above clavicle, Louis angle or estimated right atrium position).^21,22^ In patients with heart failure with preserved systolic function, the jugular vein pressure is far less studied.^23,24^ In accordance with these observations, we have also found that elevated jugular venous pressure had the best specificity (88%) of all physical findings for elevated LAP. Additionally, in patients with no elevated jugular venous pressure, but with a positive hepatojugular reflux, we were able to identify an elevated LAP in 10 out of 12 patients. However, as expected, the absence of elevated jugular venous pressure was not able to exclude elevated LAP.

#### Third heart sound

Collins et al.^25^ have studied patients with dyspnea in the emergency department and found that S3 did not improve diagnostic accuracy for ADHF, with a sensitivity of only 14.6%. Moreover, in that study, a low diagnostic accuracy (58%) for the diagnosis of ADHF when utilizing all signs together was observed. In our study, S3 was present in less than half of patients and, when present, showed a positive predictive value of 79% for LAP > 15 mm Hg. When absent, an elevation of filling pressures could not be ruled out. In addition, S3 did not add any information regarding hemodynamic status. This is in accordance with other studies.^3,26^ Of note, in our study, all physical examinations were done by a heart failure specialist. In the setting of a less experienced professional, the accuracy of physical exam (particularly S3) can be low, since studies suggest poor agreement between medical interns or residents and phonocardiographic findings.^27^ On the other hand, reasonable agreement in S3 detection has been found among professionals of heart failure clinics.^28^

#### Levels of BNP

The strongest evidence for clinical use of BNP is to discriminate the cause of dyspnea in patients admitted in the emergency department^29^ and to assess prognosis.^30,31^ For other BNP purposes, data are less clear. In the Escape Trial,^32^ the ROC curve for the performance of BNP in estimating an elevation in PCWP > 22 mm Hg showed a poor performance (AUC = 0.55). Another study with 40 critically ill patients utilizing invasive hemodynamic monitoring has shown a weak correlation between BNP and PCWP (r = 0.58).^33^ Our data were consistent with these studies, showing a weak correlation between BNP and LAP (r = 0.29). BNP was also tested for guiding treatment, because, theoretically, lowering BNP is a consequence of lowering filling pressures,^34^ but this strategy failed to show clinical benefit.^35^ In contrast, in the recent PROTECT trial,^36^ a similar strategy of guiding treatment according to amino-terminal-Pro-BNP levels against standard of care resulted in decreased incidence of events, improvement in quality of life and in cardiac remodeling. However, that trial was conducted in an outpatient setting, involving very few heart failure patients in more advanced functional classes.

In the present study, we used a cut-off point of 400 pg/mL for BNP as a marker of congestion, since this value was employed in previous studies.^14,35^ We have observed that BNP levels > 400 pg/mL had a poor prediction performance to identify elevation in LAP, similar to other physical findings or chest radiography performances when taken individually. No valuable information on filling pressures was observed when BNP levels were below 400 pg/mL. Using the AUC, we found that BNP levels of 1000 pg/mL had the best specificity to predict LAP ≥ 15 mm Hg. Therefore, we have also utilized this cut-off value in our subsequent combined analysis. Patients with moderate or severe renal impairment had higher BNP values, in our study the mean values of urea and creatinine were only slightly elevated and should not have influenced the results.

Although there is a time difference between the change in ventricular filling pressures and the corresponding change in BNP levels, this time lapse does not seem to have clinical significance. The half-life of BNP is short, about 20 minutes, and, in addition, the treatment-induced decrease in pulmonary capillary pressure leads to a rapid reduction in BNP levels (30 to 50 pg/mL/hour).

### Combining tools to estimate congestion

In patients with intermediate BNP levels (100-500 pg/mL), adding the information about the presence of S3 increases the positive predictive value from 54% to 80%.^37^ A recent study with 50 patients utilized a very similar strategy to our study, comparing a CS, BNP and a hand carried ultrasound in estimating elevation of ventricular filling pressures, but the gold standard in that study was right heart catheterization.^14^ As in ours, that study used a cut-off value for BNP > 400 pg/mL and for PCWP ≥15 mm Hg as referencing parameters. The clinical symptom score had very little predictive utility for an elevated PCWP. Combining the information of jugular venous pressure, BNP and ultrasound, the best diagnostic characteristics for predicting elevated LV filling pressure was achieved (AUC 0.98). In our study, combining the findings of physical examination with chest radiography and BNP augmented progressively the sensitivity (64%, 82% and 91%, respectively) for detecting an elevated LAP, achieving a positive predictive value of 81%, although with a poor specificity. Still, combining these tools showed a modest power in predicting high filling pressures (AUC: 0.62). Thus, ours and the study by Goonewardena et al.^14^ showed that clinical examination and BNP are not fully capable to precisely detect elevated filling pressures, and echocardiographically-derived hemodynamic assessment can reliably be incorporated into clinical practice of ADHF, avoiding the traditional invasive right heart catheterization method. The increasing utilization of hand carried ultrasound can be of great value in this area.

### Study limitations

We have used the echocardiogram as the gold-standard method for defining filling pressures instead of right heart catheterization. Nonetheless, hemodynamic echocardiogram-derived parameters are well validated in the medical literature when correlated with invasive measurements.^38-40^

We primarily use the lateral annulus to measure E/e' ratio. Although the most recent recommendations suggest using the mean of the lateral and septal annulus values, this was mainly validated in normal subjects. The most recent 2016 guideline of the American Society of Echocardiography and the European Association of Cardiovascular Imaging recognizes that at times only the lateral e’ or septal e’ velocity is available, and it is clinically valid.

In addition, we did not follow patients during the hospitalization or in the post-discharge period to observe whether the initial hemodynamic profile was compatible with the clinical course.

## Conclusions

In this study, we showed that in ADHF patients, clinical assessment alone or in conjunction with chest radiography and BNP may lead to inaccurate estimation of echocardiographically-derived hemodynamic profiling.
